# Supplementary material for: Are carbon emissions trading and green financial instruments synergistic? -Comprehensive quantitative research based on content analysis
Source: PLoS One. 2024 Mar 7;19(3):e0298601. doi: 10.1371/journal.pone.0298601 (PMC10919601; doi:10.1371/journal.pone.0298601)
Supplement: S1 Data — (ZIP) [file pone.0298601.s001.zip › Data/地级市Z_F工作报告.zip!/╕ú╜¿/┴·╤╥╩╨/└┤╫╘╣1⁄2╓┌║┼í╛┬φ┐╦╩2╛▌═°í┐_2016.docx]

各位代表：

　　现在，我代表龙岩市人民政府，向大会报告政府工作，请予审议，并请市政协委员和其他列席人员提出意见。

　　一、2015年和“十二五”时期工作回顾

　　过去一年，在省委、省政府和市委的正确领导下，我市各级政府紧紧抓住中央支持福建加快发展、支持原中央苏区振兴发展和“新古田会议”召开的重大机遇，主动适应新常态，全力以赴稳增长、调结构、促改革、惠民生，经济社会保持平稳发展。初步统计，全市地区生产总值1739亿元，增长8.9%；一般公共预算总收入269.8亿元、增长3.1%，地方一般公共预算收入124.6亿元、增长4%；全社会固定资产投资1934亿元，增长21.6%；社会消费品零售总额640亿元，增长14.2%；外贸出口160亿元，增长7.8%；实际利用外商直接投资2.7亿美元，增长11.5%；内联引资实际利用市外资金196亿元，增长10%；城镇居民人均可支配收入31049元，增长7.5%；农民人均可支配收入12939元，增长10%；居民消费价格总水平上涨1.6%；城镇登记失业率2.48%；人口自然增长率12.2‰；年度节能减排任务预计可以完成。

　　一年来的主要工作和成效是：

　　（一）全力保持经济平稳增长。出台扶持工业“十一条”，千方百计帮助企业解决资金紧张、市场开拓等难题。完善金融机构考核奖励办法，建立银担合作机制，加大金融服务实体经济力度，不良贷款率保持低位运行。及时兑现落实帮扶资金，下达工业发展专项资金8800万元，减免小微企业税费1.7亿元，为280家企业提供43亿元还贷周转资金。在产能过剩、市场低迷的大环境下，实现规模以上工业增加值574亿元，增长8.7%。龙马环卫成功上市，卫东实业、永强岩土在“新三板”挂牌。

　　（二）“项目会战年”成效明显。落实全省“三比一看”，组织开展“项目会战年”活动，全力打好项目攻坚战。省、市重点项目完成投资659亿元，占年度计划的128.3%。交通、水利等七大领域完成投资303亿元，占年度计划的110%。军民融合产业发展走在全省前列，签订合同项目27个、协议项目16个，已落地24个，共有20家企业产品供应部队、军工企业。德晖光电、上杭中宇水暖等项目开工建设，紫金山矿产资源综合利用、漳永高速、古武高速龙岩段、龙岩高速互通迁建等项目建成或投产，赣瑞龙动车新线开通。

　　（三）产业转型步伐加快。制定实施加快产业转型升级、发展智能制造、推进创业创新、支持住房消费等政策。九大重点工业产业实现产值1773亿元、增长9%。专用车年产突破1万辆、产值增长15%，光电与新材料、节能环保、生物医药等新兴产业分别增长17.2%、18%和27.3%。第三产业增加值621亿元、增长9.8%，改变了多年来滞后于经济增速的局面。电子商务异军突起，全年交易额突破150亿元。销售商品房220万平方米、增长25%，促进农民进城购房做法得到住建部充分肯定。旅游收入195亿元、增长18%，古田景区成功创建国家5A级旅游景区，冠豸山机场新开通成都、桂林、杭州等航线，旅客吞吐量突破10万人次。

　　（四）现代农业稳步发展。实现农林牧渔业总产值335亿元、增长4%。粮食单产和总产连续八年增长，林下经济产值121亿元、增长19.3%，果蔬、林竹、茶叶、花卉苗木等特色产业持续增长。评选出闽西“八大珍”、“八大鲜”，新增市级龙头企业7家，农产品加工业产值285亿元、增长9.4%。实现农村淘宝全覆盖，农产品电商交易额突破10亿元。

　　（五）新型城镇化扎实推进。调整“三区两园”管理体制，“多规合一”编制进展顺利。中心城市主干路网建设加快，兴业路一期、龙腾北路五期全面贯通，启动龙岩火车站片区开发。镇级“小城市”培育试点、“千村整治、百村示范”工程建设稳步推进，连城培田村被农业部评为“中国最美休闲乡村”。城市环境卫生、违章建筑整治得到加强，全市共拆除“两违”面积838万平方米，完成省下达任务的2.3倍。

　　（六）改革开放纵深推进。完成市、县两级政府机构改革、事业单位分类工作。在全省率先推出行政审批制度10项改革创新“组合拳”，取消、下放行政审批和公共服务事项79项，完成“三张清单”公布，全面推广“三证合一”、“一照一码”登记制度改革。龙岩公共服务网正式上线运行，行政服务中心通过全国公共服务综合标准化单位验收。全面启动城市和县级公立医院综合改革，长汀基层医改经验获2015年“中国政府创新实践奖”。完善国有企业管理体制，组建水务发展集团。不动产统一登记、农村土地承包经营权确权登记颁证试点顺利实施。成功举办第六届海峡两岸机械产业博览会暨福建龙岩专用车投资贸易洽谈会，共签约项目72个、总投资280亿元。新增境外投资企业9家、总投资14.6亿美元，创历史新高。厦龙山海协作经济区入驻企业12家、总投资39亿元。

　　（七）生态优势进一步凸显。成功创建国家森林城市，通过省级生态市考核验收。完成造林绿化23.6万亩，治理水土流失45.3万亩。全市共投入8.4亿元，关闭拆除禁养区养猪场1万多户、削减生猪134万头，标准化改造可养区规模养猪场820户。重点流域水质持续改善，中心城市内河及闽江、汀江流域水质达标率均高于目标要求。淘汰黄标车7297辆，中心城市环境空气质量优良以上天数360天、占98.6%。

　　（八）民生保障水平不断提升。22件为民办实事项目全面完成。新增城镇就业2.5万人，农村劳动力转移就业4.9万人。保障性安居工程新开工2万套、基本建成1.98万套，分别完成任务数的123.6%和209.1%。《长（汀）连（城）武（平）扶贫开发试验区发展规划》获得省政府批复，国土资源部专门出台支持我市脱贫攻坚政策。积极应对“5·19”、“7·22”等突发重大洪灾，最大限度减少灾害损失，受灾重建户基本可在2016年春节前迁入新居。北大附属实验学校正式招生，龙岩技师学院第二校区、龙岩一中分校开工建设；闽西职业技术学院通过国家骨干校评估。成立市医药卫生职业教育集团，市第一医院门诊综合楼建成使用，全省首创“互联网+分级诊疗”项目建成试运行。群众公共文化活动深入开展，文化遗产保护成效显著。圆满承办第一届全国青年运动会举重比赛，龙岩籍运动员在羽毛球、蹦床、举重、射击项目的国际大赛中获得4枚金牌。安全生产“四项指数”全面下降，食品药品安全有效保障，群众安全感率、平安建设知晓率、执法工作满意率均居全省首位。村级组织换届全面完成。龙岩市和武平县获得全国文明城市提名城市。援藏援疆工作扎实推进。国防动员和后备力量建设成效明显，龙岩市和连城县、上杭县通过全国“双拥模范城（县）”考评验收。

　　（九）自身建设得到加强。深入开展“三严三实”专题教育，严格落实中央八项规定精神，贯彻落实市委《关于在全市党员干部中纠正不良习气树立清风正气的意见》，持续改进作风。政府系统“三公”经费支出下降18%，完成机关办公用房清理整改。自觉接受市人大、市政协和社会各界的监督，共办理人大代表建议285件、政协提案564件次，办复率均达100%。政府与法院、检察院、工会互动联系机制不断完善，依法行政、机关效能建设持续深化，行政监察和审计监督力度加大，反腐倡廉工作扎实推进。

　　2015年各项任务的完成，标志着我市“十二五”规划任务基本实现。“十二五”时期，是龙岩发展迎来重大历史机遇并取得明显进步的五年。中央作出了支持福建加快发展、支持原中央苏区振兴发展等一系列重大决策部署，“新古田会议”在我市胜利召开，给了我们极大的鼓舞。经济发展进入新常态，面对加快发展与转型升级的双重压力，我们在市委的坚强领导下，团结一心，迎难而上，积极应对各种风险挑战，经济社会发展迈上新台阶，新的增长点加快孕育，为长远发展奠定坚实基础。

　　五年来，综合实力明显增强。地区生产总值、全社会固定资产投资、财政收入、社会消费品零售总额、外贸出口、城乡居民收入等主要指标实现两位数增长，人均地区生产总值突破1万美元。全社会固定资产投资累计完成6600亿元，是“十一五”时期的3.7倍。三次产业结构由13.0:53.3:33.7调整为11.5:52.8:35.7。高新技术产业增加值占GDP比重提高4.3个百分点、达10.9%。

　　五年来，城乡面貌深刻变化。高速公路通车里程660公里、是2010年的2倍，新增快速铁路171公里，在全省山区市中率先实现县县通高速、率先开通动车。永定撤县设区，中心城市建成区面积扩大15平方公里、达53平方公里，成功创建国家园林城市。全市常住人口城镇化率提高7.1个百分点、达52.1%。建成一批美丽乡村，基本实现村村通广播电视、通宽带。

　　五年来，生态环境巩固提升。森林覆盖率达77.9%，比2010年提高4.8个百分点，保持全省首位。治理水土流失面积256万亩，“长汀经验”在全国总结推广，成为南方红壤区治理的典范。完成高标准基本农田建设24万亩，连续16年实现耕地占补平衡。水环境进一步好转，中心城区内河水质连续两年100%达标。淘汰落后产能、节能减排成效明显。

　　五年来，发展活力持续增强。行政服务标准化成为国家级试点，基层医改“长汀模式”、林权改革“武平经验”在全国、全省推广，新型职业农民培育和“三农”综合保险在全国开创“龙岩模式”。民营经济更加活跃，民间投资占全社会投资的四分之三。成功创建国家级经济技术开发区、高新技术产业园区、可持续发展实验区、全国科技进步先进市，与厦门共建山海协作经济区，发展平台进一步拓展。参照执行西部地区政策共落实103项，获得中央补助410多亿元。

　　五年来，民生事业长足进步。民生支出超过720亿元，占全市公共预算的74.9%。减贫11.5万人，完成造福工程危房改造3.7万户，建成保障性安居工程3.7万套，解决170.6万农村人口饮水安全问题，精准扶贫“九到户”举措得到国家和省里肯定并推广。新罗、永定、上杭通过“义务教育发展基本均衡县”国家级验收，全面完成中小学标准化学校建设，高等教育、职业教育加快发展。新增医疗机构床位4940张，人均基本公共卫生服务经费标准由15元/人提高到40元/人。城乡居民社会养老保险基础养老金实现倍增。全民健身、竞技体育协调发展。“平安龙岩”建设持续深化。“六五”普法通过省级验收。人事、统计、民族宗教、外事侨务、库区移民、水文、气象、地震、地灾防治、人防、档案、地方志、科普、邮政管理、无线电管理、老年人、妇女儿童、青少年、残疾人以及慈善事业等工作进一步加强。

　　各位代表，成绩来之不易。这是省委、省政府和市委正确领导、全市人民奋力拼搏及各方面大力支持的结果。我代表龙岩市人民政府，向全市人民，向人大代表、政协委员、各民主党派、工商联、各人民团体、无党派人士，向离退休老同志、在外乡亲和社会各界人士，向中央和省直驻岩机构、驻岩部队、武警官兵、公安民警，向关心支持龙岩发展的港澳台同胞、海外侨胞和国际友人，表示衷心的感谢！

　　在看到成绩的同时，我们也清醒地认识到存在的困难和问题，主要是：在经济下行的大背景下，我市传统产业竞争力减弱，经济增长新动力不足，调结构、促转型任务艰巨，稳增长的压力较大；部分企业自主创新能力不强，产品市场竞争力较弱，生产经营困难；城乡之间、县域之间发展不平衡，农村基础设施还比较薄弱；社会保障和教育、卫生等社会事业发展与人民群众的期待还有差距，公共安全还存在不少隐患，脱贫攻坚任务依然艰巨；政府服务效能和水平有待进一步提高，不作为、慢作为、乱作为和消极腐败等现象在某些地方、某些领域仍有发生。对此，我们将坚持问题导向，采取有力措施认真加以解决。

　　二、全面实施“十三五”规划

　　这次提请大会审议的《龙岩市国民经济和社会发展第十三个五年规划纲要（草案）》，明确提出了“十三五”发展的指导思想、发展目标、战略任务和保障措施，描绘了今后五年经济社会发展的宏伟蓝图。

　　“十三五”发展的指导思想是：高举中国特色社会主义伟大旗帜，全面贯彻党的十八大和十八届三中、四中、五中全会精神，以马克思列宁主义、毛泽东思想、邓小平理论、“三个代表”重要思想、科学发展观为指导，深入贯彻习近平总书记系列重要讲话精神，坚持“四个全面”的战略布局，坚持创新、协调、绿色、开放、共享的发展理念，坚持发展第一要务，以保持经济稳定较快增长为目标，以转型升级为主线，以提高经济发展质量和效益为中心，创新开放“双轮驱动”，生产生活生态“三生融合”，城市乡村“两全其美”，努力打造东进沿海、西连腹地的生态型经济枢纽，建设机制活、产业优、百姓富、生态美的新龙岩。

　　“十三五”发展的主要目标是：综合实力迈上新台阶，地区生产总值年均增长9%左右，增长速度高于全省平均水平，提前实现地区生产总值和城乡居民人均可支配收入比2010年翻一番。产业转型升级实现新突破，三次产业结构调整为8.5:48.5:43.0，发展质量和效益显著提高。城乡建设呈现新面貌，城镇化布局和形态更加优化，户籍人口城镇化率达到48%。改革开放构建新格局，重要领域和关键环节改革取得决定性成果，开放水平和质量进一步提高。生态文明形成新优势，创建国家生态文明建设示范市，森林覆盖率保持全省首位。人民生活再上新台阶，公共服务更加均等、优质，覆盖城乡居民的社会保障体系基本形成。

　　“十三五”发展的重点任务概括起来主要是：

　　（一）创新发展，打造产业升级版。实施创新驱动战略，推进大众创业万众创新，增强发展内生动力。深入实施“中国制造2025”行动计划，大力发展先进制造业，转变农业发展方式，推动现代服务业大发展，构建产业新体系。突出军民融合产业发展、专用车之城、中国绿色矿都、“互联网+”、品牌农业，培育发展“三个五”产业集群，推动经济从量的扩张向质的飞跃，探索一条富有龙岩特色的产业转型升级之路。

　　（二）协调发展，提升发展均衡性。推进以人为核心的新型城镇化，建设新型城镇体系。推进城乡基础设施一体化，增强交通、能源、水利、信息、防灾减灾等保障能力。实施精准扶贫、精准脱贫，到2018年，现行国定扶贫标准扶贫对象全部脱贫；到2020年，省定扶贫标准扶贫对象全部脱贫，实现长汀、连城、武平3个省级扶贫开发重点县及31个市级扶贫开发重点乡镇全部摘帽，380个贫困村、所有贫困户全部脱贫。弘扬社会主义核心价值观，推动文化体制改革、文化事业惠民和文化产业发展，传承创新闽西特色文化。

　　（三）绿色发展，建设生态文明先行示范区。落实主体功能区布局，加快形成生产、生活、生态融合互动的国土空间体系，基本建成生态安全保障体系。深入实施水、大气、土壤污染防治行动计划，加强环境保护和生态修复，促进资源节约和高效利用，推动低碳循环发展。健全生态文明制度体系，提高公民节能、节水、节材等意识，建设绿色、低碳、可持续发展的生态型城市。

　　（四）开放发展，增强发展活力。推进行政审批、国资国企、商事登记制度等重点领域改革，建设阳光高效的服务型政府，优化发展环境。融入21世纪海上丝绸之路核心区建设，对接福建自贸区建设，更加积极有效利用外资，推动外贸转型升级，鼓励矿业、机械、环保等优势企业加快“走出去”。提升区域协作水平，推动龙台深度融合，深化龙港澳侨合作，加强国际友城交流，拓展对外开放新空间。

　　（五）共享发展，增进民生福祉。坚持普惠性、保基本、均等化，推进和谐包容发展，让人民群众有更多获得感、幸福感。促进教育事业优质化，完善医疗卫生服务体系，健全覆盖城乡的基本公共服务体系。着力提高城乡居民收入，推动更高质量的就业。建成覆盖城乡居民的社会保障体系，加强社会救助和社会福利，均衡发展机构养老、社区养老、居家养老等多样化养老服务。全面提升社会治理能力，推进平安龙岩、法治龙岩、诚信龙岩建设，创建全国文明城市，构建公平正义、和谐稳定的社会环境。

　　三、扎实做好2016年工作

　　今年是“十三五”的开局之年，也是推进结构性改革的攻坚之年。我们既要看到经济环境复杂严峻、下行压力较大，面临各种风险和挑战；更要看到经济发展长期向好的趋势没有变，结构调整优化的前进态势没有变，我市还面临中央和省里加大对苏区老区支持力度的重大机遇。我们将进一步坚定发展信心，深入贯彻五大发展理念，坚持稳中求进总基调，推进供给侧结构性改革，着力稳增长、调结构、强动力、惠民生、防风险，打好项目落地、脱贫攻坚、生态环保三大战役，保持经济平稳较快发展，在优化结构、补齐短板上取得新进展，在深化改革、开放合作上取得新突破，在民生事业、社会保障上取得新提升，为“十三五”发展开好局、起好步。

　　今年发展主要预期目标是：全市地区生产总值增长9%左右；一般公共预算总收入增长6%，地方一般公共预算收入增长6%；全社会固定资产投资增长18%；社会消费品零售总额增长14%；外贸出口增长2.5%，实际利用外商直接投资增长6%，内联引资实际利用市外资金增长7%；城镇居民人均可支配收入增长9%，农民人均可支配收入增长9%；居民消费价格总水平涨幅控制在3%左右；城镇登记失业率控制在4.2%以内；完成省下达的节能减排降碳任务。

　　为实现上述目标，重点抓好以下九个方面工作：

　　（一）从供给侧发力，加快产业转型升级

　　集聚发展重点产业。加快壮大有色金属、机械、商贸物流等优势产业，提升烟草、建材、能源精化、纺织等传统产业，培育光电与新材料、节能环保、新能源等新兴产业，优化存量、创造增量，全年规模以上工业增加值增长8.5%。围绕打造“专用车之城”，积极引进战略投资者、专用车优质企业，推进专用车研究中心建设，大力发展城乡保洁、应急抢险、防恐防爆、冷链物流等各类专用车，推进海德馨应急专用车及防恐防暴装备、新龙客车等项目建成投产，开工建设新龙马枭龙越野车生产基地、龙马环卫装备扩建、侨龙专用车扩建等项目。围绕打造“中国绿色矿都”，发展与矿业配套的机械、矿山工程、现代农业、金融、物流、配套服务等产业，开工建设紫金山罗卜岭铜矿采选等项目，建成投产紫金山金铜矿浮选厂、金龙稀土年产6000吨稀土特种金属及合金等项目。加快发展光电与新材料产业，突出抓好武平新型显示和终端产业园、连城光电产业园、德泓光电产业园建设，推进正德光电新材料生产二期、达米拉智能电视及系列家用电器生产等项目加快建设，促进尚方智能终端显示多媒体等项目开工建设、中晶蓝宝石图形化衬底等项目建成投产。实施新一轮技术改造，突出抓好80个重点工业技改项目，推动传统产业加快改造提升。

　　增强企业竞争力。开展降低企业成本行动，进一步规范和减免涉企收费，强化融资、用电等服务保障，降低企业制度性交易成本、税费负担、财务成本，提高市场竞争力。积极帮助企业拓展市场，加快去产能去库存步伐，稳定企业生产。实施“一业一策”、“一企一策”，重点扶持今后三年新增产值50亿元或今后五年新增产值100亿元以上龙头企业，推动有市场、有潜力的中小企业上规模、上水平，全年新增亿元以上企业30家、规模以上工业企业60家。积极稳妥推进优胜劣汰，支持企业开展兼并重组。

　　做强做优园区平台。优化产业发展布局，推动园区差异化、特色化发展。理顺龙岩经济技术开发区（高新区）管理机制，着力打造先进制造业、高新技术产业集聚平台。加快厦龙经济区基础设施建设，主动承接沿海产业转移，着力打造新型交通装备制造基地。加强县域工业园区建设，进一步完善基础设施，健全配套服务功能，促进产城融合发展。引导产业向专业园区集聚，重点做大金铜、稀土、不锈钢等特色产业园区。推动厦龙经济区、上杭蛟洋工业集中区、晋江（长汀）工业园升格为省级工业园区。

　　（二）打好项目落地战役，增强投资拉动力

　　扩大有效投资。持续推进2014～2018重大投资项目，续建项目257个、新开工项目56个，完成投资570亿元以上。安排重点项目300个，年度投资607亿元，增长18%以上。突出抓好投资5亿元以上或新增产值10亿元以上的工业重大项目，完成工业投资1000亿元以上。抓好交通、能源、市政、环保、水利、信息通信等领域重大项目建设，基础设施完成投资520亿元以上。全社会固定资产投资完成2280亿元以上。

　　加强项目接续。围绕打造“三个五”产业集群和增加公共产品、公共服务，加强重大项目策划、储备和实施，形成“谋划一批、签约一批、开工一批、投产一批、增资一批”的项目滚动发展格局。精准对接国家专项建设基金投向，实施政府投资项目三年滚动投资计划。做深做细前期工作，促进项目早落地早开工。

　　强化服务保障。完善领导挂钩重大项目和“一月一协调、一季一督查”推进落实机制，建立重特大项目“绿色通道”，加大用地、用林、审批、征迁等服务，保障项目顺利实施。创新政银企担风险共担机制，推动银行更好支持项目建设。创新投融资机制，运用发债、资产证券化等多种方式融资，支持重点领域建设项目开展股权和债权融资。大力推广政府购买服务、公私合作等模式，引导社会资本参与基础设施和医疗卫生、养老服务等领域建设，进一步激活民间投资。

　　（三）着力融合创新，培育发展新动能

　　推进军民融合产业发展。持续深化“新古田会议”后续效应，进一步畅通“军转民”、“民参军”渠道，探索军地“产—军—学—研”融合发展新模式，争创国家级军民结合新型工业化产业示范基地。拓展军民技术对接、股权合作等多种合作形式，着重加强在专用车、光电、冶金新材料、文化旅游、影视等领域的合作。推动干部双向挂职交流，密切军地沟通联系。

　　发展互联网经济。推进移动互联网、云计算、大数据、物联网与我市现有产业结合，催生发展新产业、新业态、新模式，培育新的产业接续和支撑力量。继续深化与阿里巴巴、京东等电商平台的合作，推动美团网、今日头条、雪球网等龙岩籍互联网企业家回龙岩设立区域中心。加快长汀、武平全国电子商务进农村综合示范县和连城、漳平省级农村电子商务示范县建设，打造10个电子商务基地（园区）、培育6家电子商务示范平台（企业）。重点推动传统商贸流通和旅游业线上线下深度融合，大力发展网络零售和在线旅游，电子商务交易额突破200亿元。

　　推动服务业优质发展。加大服务业龙头企业培育力度，新增限上企业200家以上。组建文化旅游发展集团，加大红色、客家、生态等资源整合开发力度，提升古田、土楼、冠豸山、长汀历史文化名城等重点景区开发建设水平，完善配套服务设施，开发特色产品，带动观光度假、体育健身、教育培训、文化创意等行业发展。大力发展现代物流业，加快龙岩公路港、中国（龙岩）农产品物流交易城等项目建设，争取设立龙岩陆地港保税物流中心，探索设立区域性分拣中心，打造区域性物流节点城市。打通保障性住房与安置房、商品房的通道，支持住房刚性需求，发展住房租赁市场，加快消化库存，促进房地产市场健康稳定发展。发挥生态优势，推进医疗卫生与养老服务相结合，加快发展新型健康养老产业，抓好东肖养生养老产业园等项目建设。落实和完善鼓励消费政策，促进批发零售、金融保险、交通运输等传统服务业提升做强。

　　推进创业创新。强化企业创新主体，支持开展科技、管理、服务、品牌和商业模式等创新，带动全社会创新。实施“科技小巨人行动计划”，用好龙岩紫荆创新研究院、厦龙工程技术研究院和科技企业孵化器等创新平台，突破关键共性技术。促进工业化、信息化“两化”融合，加快发展智能制造，提升装备数控化水平。设立产业引导基金，鼓励风投、创投、社会资金设立子基金，支持发展新兴产业和先进制造业。实施大学生返乡创业计划、农村青年创业富民行动，支持奇迈创客等新型众创空间建设，培养引进一批科技创新人才和研发团队，充分激发多元创业创新主体的活力。

　　（四）突出特色高效，大力推进农业现代化

　　培育特色品牌农业。加快发展林下经济和竹业、茶叶、花卉苗木等特色产业，实现农产品加工业产值300亿元以上。加强地方特色品种的保护与开发，以闽西“八大干”、“八大珍”、“八大鲜”和特色饮品为重点，进一步壮大规模、打响品牌。支持智能温控、水肥一体化等设施农业项目，推进农产品标准化生产，建立完善产品质量安全可追溯平台，提高农产品供给质量。

　　提高农业生产能力。稳定粮食生产，落实粮食播种面积271.5万亩。推进高标准基本农田建设，加强水库除险加固工程建设，加快实施汀江、九龙江防洪工程、烟草水源工程，抓好万安溪引调水工程、连城福地水库、长汀荣丰水库、新罗坪坑水库等项目。推广农业“五新”技术，支持农业信息化试点示范。培育新型农业经营主体，扶持发展农民合作示范社和示范家庭农场，新颁证认定新型职业农民千名以上。

　　拓展农业发展空间。推进种养加一体、一二三产融合发展，扶持农村电商加快发展，释放农业增长潜力。推进农村土地承包经营权确权登记颁证，促进农村土地经营权有序流转，发展多种形式适度规模经营。深化集体林权制度、生态公益林管护体制改革，全面开展国有林场改革。创新农业投入机制和财政、金融支农方式，提升“三农”综合保险水平，引导更多社会资金投入农业农村。

　　（五）提高新型城镇化质量，建设和谐宜居城乡

　　增强中心城市带动功能。按照“一市两区”发展格局，统筹推进重点产业、基础设施规划建设，推动永定区加快融入中心城市。加快火车站片区开发。推进中心城市“一环一轴四纵五横”主干路网建设，开工建设龙岩高速东环线，建成西外环、莆永高速龙岩北互通连接线，打通金鸡路、犀牛路排头段等“断头路”。推进中心城区范围内高速公路市政化管理，促进高速公路与城市主干道路互联互通。加强人行通道、公共停车场和地下综合管廊建设，新增公共停车位1500个以上。推广应用交通“消费一卡支付”，提升公交便民服务水平。开展“海绵城市”建设试点，完善雨污分流系统，提升城市排水防涝能力。

　　推进区域协调发展。编制实施“多规合一”，加强城市设计。加快建设漳州天宝至龙岩蛟洋高速改扩建、南三龙铁路等项目，开工建设浦梅铁路建宁至冠豸山段，推进龙岩经梅州至龙川快速铁路前期工作，新开通冠豸山机场至北京航线，拓宽对外连接通道。推进“一市两区”及古田、土楼旅游区一体化发展，建成中心城区至龙岩高新区城际快速通道。抓好国省干线、县乡道路和农村公路提升改造。继续推进高陂、古田等8个“小城市”培育试点建设，实现连片融合发展。全面实施居住证制度，推进基本公共服务向常住人口覆盖，促进农民工加快融入城市。

　　推进宜居城乡建设。实施道路畅通、旧城改造、市政提升、东肖新城开发、农村环境整治、住宅安居等“六大工程”，打造城建“十大亮点”，彰显城市特色和魅力。创新城市治理方式，加强城市精细化管理，推进物业管理规范化，开展燃油助力车专项治理，大力整治违法建设、违章搭盖、乱倒垃圾弃土等突出问题。加强城乡集贸市场建设管理，实施标准化建设改造8个以上。推进家园清洁行动，强化农村环境整治，全面完成130个省级“千村整治、百村示范”项目和市级34个美丽乡村试点建设，建立完善农村环卫保洁长效机制，美化乡村环境。加强历史文化名城名镇名村、传统村落、古民居保护，守住历史风貌，留住民俗风情。

　　（六）打好生态环保战役，推进绿色低碳发展

　　加强生态保护修复。成功创建省级生态市，推进国家生态文明建设示范市创建工作。继续实施水土流失治理工程，治理面积43万亩，治理崩岗100个，实施坡耕地综合整治1000亩。加强森林资源保护，造林绿化8.8万亩，封山育林37.1万亩。实施山林田湖生态保护和修复工程，科学划定并严守生态红线。加大植被修复保护，增强流域水源的涵养功能。强化黄岗水库等饮用水源地保护。加强地质灾害防治。

　　推动环境质量改善。严格执行“河长制”，抓好江河湖库水葫芦、永定洪山石板材、养殖业污染、小水电四大专项整治，全面关闭拆除禁养区养猪场，完成可养区规模养猪场标准化改造提升，促进流域水质根本性好转。强化矿山生态环境恢复治理，升级改造小煤矿11家，关闭14家。对造纸、化工、建材等重点排污企业实行全行业排污总量控制，加快建设乡镇污水处理设施及管网、危险废物综合处置设施及填埋场。推动中心城区污染企业、风险企业搬迁关闭，加快火电、钢铁、水泥等重点行业烟气脱硫、脱硝和除尘设施建设，强化建筑施工、道路扬尘和机动车尾气污染防治，继续淘汰“黄标车”，中心城市空气质量优良以上天数保持在97%以上。

　　提高资源利用效率。严格环境准入，实行能源和水资源消耗、建设用地等总量和强度双控制度。实施节能技改工程和循环经济重点项目，推动工业园区、企业开展循环化改造、清洁生产。实施燃煤锅炉节能环保综合提升工程，推动水泥、火电等重点用能行业开展节能量交易工作。推动龙雁经济开发区、上杭蛟洋工业集中区创建国家级、省级循环经济园区。引导群众树立绿色低碳的消费模式和生活方式。

　　（七）深化改革开放，打造良好发展环境

　　突出重点领域改革。动态调整“三张清单”，全面放权县（市、区）和龙岩经济技术开发区，推行网上并联审批。深化商事登记制度改革，强化事中事后监管。全面深化公立医院和基层医疗卫生改革，重点加快分级诊疗、控费管理、社会力量办医和社区卫生服务中心建设。推进市属国有企业分类改革、整合发展，推动国有资产资本化、证券化，实现国有资产优质化。加快结转资金清理，竞争分配专项资金，提高财政资金使用效益。全面实施政府性债务限额管理，创新债务风险应急预警管理机制。支持企业股改上市，力争1家以上企业上市、5家以上企业在“新三板”挂牌。抓好公务用车、标准化、统计方式等改革。

　　加快开放型经济发展。对接福建自贸区、21世纪海上丝绸之路核心区建设，通过紫金矿业“带路”，带动更多企业“走出去”拓展海外市场。完善龙岩陆地港口岸功能，提升外贸通关便利化水平。调整外贸出口结构，提高资本和技术密集型产品的出口比例，培育出口新增长点。整合招商资源，完善招商机制，用好“6·18”、“9·8”及“11·8”等经贸平台，提高招商引资、项目对接的实效，全力突破重特大生产性项目。

　　深化对外交流合作。加强海峡两岸交流基地建设，促进龙台产业深度对接，拓展教育、文化、科技等领域的交流合作。扩大龙港龙澳合作和国际友城交流，做好华侨华人和华裔新生代工作，密切与异地商会的联系，拓宽引资引智渠道。深化闽西南五市和闽粤赣十三市合作，积极推动厦漳泉龙同城化发展。继续抓好对口援藏、援疆工作。

　　（八）打好脱贫攻坚战役，开创扶贫开发新局面

　　实施精准帮扶。实施精准扶贫行动计划，深化精准扶贫“九到户”措施，精准施策，分类推进，确保扶贫对象到户到人，全年减贫3万人、减贫率23.3%。组织实施《长（汀）连（城）武（平）扶贫开发试验区发展规划》，支持在金融服务、农村产权制度、城乡一体化发展等领域先行先试。加大对380个贫困村和521个革命基点村、51个少数民族村倾斜支持力度。

　　健全扶贫机制。健全领导挂钩、对口帮扶机制，动员社会力量参与扶贫，建立多元主体的社会扶贫体系。集中力量实施一批基础设施、生态建设和民生工程项目，改善贫困地区生产生活条件。推行产业扶贫、电商扶贫、金融扶贫，支持贫困对象发展特色种养、乡村旅游、农村电商等增收项目，增强内生“造血”能力。完善贫困人口进退机制，逐户销号、脱贫到人。

　　强化保障兜底。提高农村低保标准，加大对困难群众的医疗救助、临时救助力度，对无法依靠产业扶持和就业帮助脱贫的家庭实行政策性保障兜底。加大移民生态搬迁力度，全年安排造福工程危房改造4万人，新建16个省级、30个市级集中安置区，加强集中安置区基础设施和公共服务配套建设。

　　（九）持续改善民生，共享发展成果

　　筑牢保障安全网。实施好25项为民办实事项目。创业带动与促进就业并举，解决好高校毕业生、城镇困难群众、农民工等重点人群就业，全年新增城镇就业2.2万人、农村劳动力转移就业4.7万人。新建保障性安居工程1.2万套，完善分配和运营监管机制。深化机关事业单位养老保险制度改革。推动社会保障从“制度全覆盖”向“人员全覆盖”转变，探索建立各种社会保险相互衔接、协调发展的机制，完善失地农民保障制度，提供多层次保障需求。发展社会福利、慈善和残疾人事业，保障妇女儿童、青少年、老年人合法权益。

　　加快发展社会事业。推进“义务教育发展基本均衡县”创建工作，确保武平县通过国家级验收。抓好农村薄弱学校改造、城区中小学校扩容项目，新建公办幼儿园10所，促进教育资源均衡配置。支持龙岩学院争创省级示范性应用技术型本科院校、闽西职业技术学院建设以工科为主的国家骨干职业院校，推动龙岩技师学院纳入高校序列，培育我市产业急需的实用型人才。支持发展民办教育。实施中医药服务能力提升工程，加强薄弱乡镇卫生院建设，完成100所“公建民营”规范化村卫生所建设，培养100名农村医学专业人才。全面实施两孩政策，完善计划生育服务管理机制。完善公共文化服务体系，推进国家级闽西客家文化生态保护实验区建设，规划建设工人文化宫。实施广播电视节目无线数字化覆盖工程和数字微波网改造。加强全民健身场地设施建设，发展体育培训、体育旅游、特色体育用品等体育产业，承办好2016年全国举重锦标赛暨奥运会选拔赛。

　　推进社会治理创新。深入开展全国文明城市创建工作。深化平安龙岩建设，完善城乡社区网格化服务管理平台，健全社会治安防控体系，提高对突发事件的应急处置能力，加大对各类违法犯罪的综合治理力度，维护社会和谐稳定。加强安全生产标准化建设，持续开展重点领域专项整治和打非治违专项行动，坚决遏制重特大安全事故发生。深入推进治理“餐桌污染”、建设“食品放心工程”，开展食品药品安全示范市三年行动，确保群众饮食用药安全。加快社会信用体系建设，建成市级公共信用信息平台。健全应急处置协调机制，有效防范和化解金融风险。启动“七五”普法工作，健全公共法律服务体系，推进法治龙岩建设。鼓励开展志愿服务活动，培育一批志愿服务品牌。抓好新一轮全国“双拥模范城”创建工作，加强国防动员、国防建设、国防教育和人民防空。

　　四、加强政府自身建设

　　我们将牢记政府前面“人民”二字，始终坚持为人民服务、对人民负责，突出忠诚、干净、担当，自觉践行“三严三实”要求，适应新常态，展现新作为，建设法治政府、廉洁政府和服务型政府。

　　忠诚履职。坚定理想信念，忠于党、忠于闽西发展事业。自觉维护市委权威，在市人大及其常委会、市政协及社会各界的监督下，以更加昂扬的斗志推进闽西苏区改革发展。把为闽西人民谋发展增福祉作为最大的责任，始终把群众冷暖忧乐放在心头，让发展成果更多更公平惠及全市人民。

　　清正廉洁。严格落实党风廉政建设责任制，认真执行《中国共产党廉洁自律准则》《中国共产党纪律处分条例》，严格落实中央八项规定精神，严格按照法定权限和程序行使权力、履行职责。坚持用制度管权、管钱、管事，加强对公共资金、公共资源、国有资产的监管。坚持民主集中制，科学决策、民主决策、依法决策，努力实现干部清正、政府清廉。

　　敢于担当。面对经济下行的压力敢于拼搏，面对深化改革中的矛盾敢于碰硬，面对产业变革带来的新模式敢于创新突破，决不囿于一时困难和问题而缩手缩脚。只要是对龙岩发展有利的事、对苏区人民有益的事，敢管敢抓、主动作为，担起应尽的责任，付出加倍的努力，做出更大的成绩。

　　实干高效。深入推进简政放权、放管结合、优化服务，加快转变政府职能。加强机关效能建设，推行“马上就办”，提高工作效率和服务质量。加大治庸治懒治散力度，严格绩效管理和行政问责，进一步提高政府执行力。

　　各位代表！“十三五”美好蓝图已经绘就，决战决胜全面建成小康社会的号角已经吹响！让我们更加紧密地团结在以习近平同志为总书记的党中央周围，在中共龙岩市委的坚强领导下，凝心聚力，真抓实干，为建设机制活、产业优、百姓富、生态美的新龙岩而努力奋斗！
